# Role of Complement System in Kidney Transplantation: Stepping From Animal Models to Clinical Application

**DOI:** 10.3389/fimmu.2022.811696

**Published:** 2022-02-25

**Authors:** Ruochen Qi, Weijun Qin

**Affiliations:** Department of Urology, Xijing Hospital, Fourth Military Medical University, Xi’an, China

**Keywords:** complement activation, kidney transplantation, ischemia–reperfusion injury, delayed graft function, T-cell-mediated rejection, antibody-mediated rejection, eculizumab, C1-INH

## Abstract

Kidney transplantation is a life-saving strategy for patients with end-stage renal diseases. Despite the advances in surgical techniques and immunosuppressive agents, the long-term graft survival remains a challenge. Growing evidence has shown that the complement system, part of the innate immune response, is involved in kidney transplantation. Novel insights highlighted the role of the locally produced and intracellular complement components in the development of inflammation and the alloreactive response in the kidney allograft. In the current review, we provide the updated understanding of the complement system in kidney transplantation. We will discuss the involvement of the different complement components in kidney ischemia–reperfusion injury, delayed graft function, allograft rejection, and chronic allograft injury. We will also introduce the existing and upcoming attempts to improve allograft outcomes in animal models and in the clinical setting by targeting the complement system.

## Introduction

Kidney transplantation is the preferred treatment for patients with end-stage renal diseases (ESRDs), which greatly improves their quality of life. The established surgical procedure and the development of immunosuppressive therapy have largely reduced the perioperative complications. However, long-term survival of kidney allografts remains challenging. Kidney ischemia–reperfusion injury (IRI), delayed graft function (DGF), T-cell- and B-cell-mediated rejection, and chronic allograft injury could all contribute to graft loss. The current perioperative induction therapy and immunosuppressive agents mainly target the activation of T cells to eliminate T-cell-mediated rejection (TCMR). However, growing evidence has shown that the complement system, part of the innate immune response, is also activated in kidney transplantation. In the current review, we will first introduce the updated knowledge on the complement system, including their components, receptors, and regulators. Then, we will discuss the roles of the different complement factors in kidney IRI, DGF, allograft rejection, and chronic allograft injury. We will also discuss the existing and upcoming therapies targeting the complement components in the scenario of kidney transplantation.

## Overview of the Complement System

**Figure 1:**
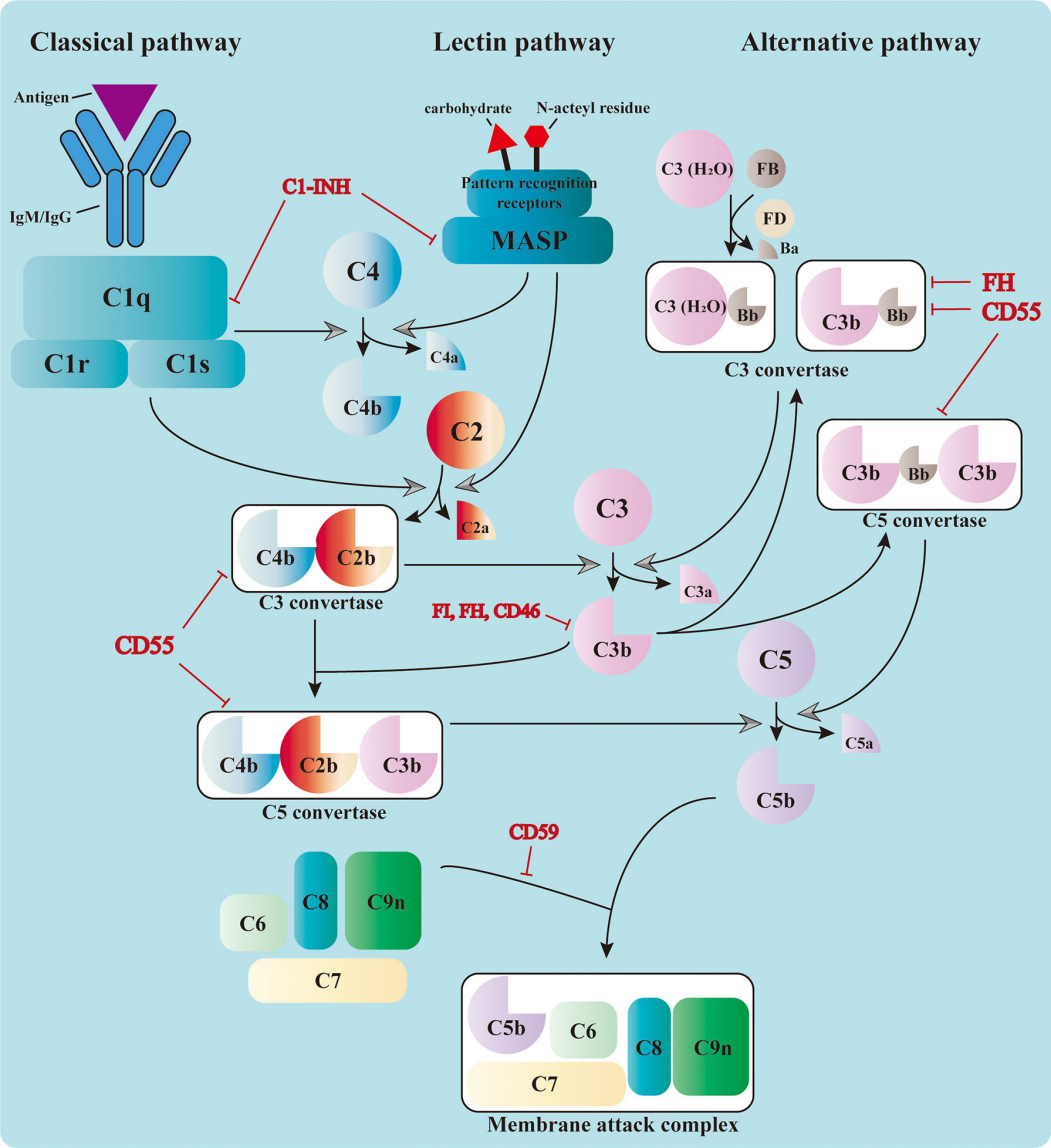
Activation of the complement cascade. The complement system could be triggered by the classical pathway, the lectin pathway, and the alternative pathway. The classical pathway is activated by the binding of C1q to the immunocomplex, which leads to the formation of the C1 complex. The latter cleaves C4 and C2 and assembles the C3 convertase, C4b2b. The serine proteases of the lectin pathway, mannose-binding lectin-associated serine proteases (MASPs), are activated by the recognition of carbohydrate and *N*-acteyl residues by the pattern recognition receptors. They also cleave C4 and C2 and form the same C3 convertase. The C3 convertase in the alternative pathway includes the initial C3(H_2_O)Bb and the latter C3bBb in the amplification phase. All three pathways converge at the cleavage of C3. The cleaved fragment C3b further adds to the C3 convertases and forms the C5 convertases (C4b2b3b or C3bBb3b), which cleave C5 into C5a and C5b. The latter leads to the formation of the membrane attack complex (MAC) together with C6, C7, C8, and C9. The complement cascade is also regulated by a variety of membrane-bound and plasma proteins to avoid uncontrolled activation.

The complement system consists of over 50 circulating, locally expressed, membrane-bound, and intracellular proteins (1). The complement factors, their cleaved fragments, corresponding receptors, and a wide range of regulators serve as integral parts of the innate immune response. Complement proteins circulating in the serum are mostly synthesized in the liver and are activated by a series of serine proteases in a cascade manner (2, 3). The cascade can be triggered by three different pathways: the classical pathway (CP), the alternative pathway (AP), and the lectin pathway (LP) (4, 5). Different pathways converge at the formation of the C3 convertase, which leads to the assembly of the C5 convertase and C5b-9, also known as the membrane attack complex (MAC) (3) (**Figure 1**).

### Initiation Phase

Three pathways adopt different strategies in sensing the activation signals (6–8). Priming of the CP relies on the recognition of immunocomplexes [antigen bound to immunoglobulin M (IgM) or immunoglobulin G (IgG)] by C1q, which leads to the sequent activation of C1r and C1s. The latter is responsible for the cleavage of C4 and C2 into small fragments (C4a and C2a) and large ones (C4b and C2b). Large fragments are further deposited on the cell surface and form the C3 convertase C4b2b (1). The LP shares the same C3 convertase with the CP; however, it is activated through the recognition of bacterial surface carbohydrate or *N*-acteyl residues by pattern recognition molecules, including mannose-binding lectin (MBL), ficolins, and collectins (9). This leads to the activation of MBL-associated serine proteases (MASPs), which can also cleave C4 and C2 and prompt the formation of C3 convertase. The activation of the AP is different from that of the other two. It relies on the spontaneous low-rate hydrolysis of the circulating C3 molecule in the fluid phase, a process known as “tickover.” The resulting C3(H_2_O) binds to factor B (FB) to form C3(H_2_O)Bb with the cleavage of factor D (FD). C3(H_2_O)Bb is the initial C3 convertase of the AP, which produces more C3b in solution. These C3b can be deposited on nearby surfaces and bind FB to form C3bBb, the amplification C3 convertase of the AP (10). This process is accelerated and amplified by the presence of pathogen surfaces and properdin, which prolongs the survival of C3b from inactivation (11). Moreover, the C3b produced from the CP and LP serves as the platform for the new AP C3 convertases and forms a positive feedback loop.

### Activation Phase

The formation of C3 convertase initiates the activation cascade of the complement system. C3 convertase (C4b2b or C3bBb) cleaves C3 into C3a and C3b, the latter of which adds to the C3 convertase and assembles the C5 convertase (C4b2b3b or C3bBb3b) (12). The C5 convertase cleaves C5 and leads to the production of C5a and C5b, the latter of which contributes to the assembly of MAC together with C6, C7, C8, and C9 (13). The MAC penetrates and forms 10-nm pores on the cell membrane, resulting in fluid and ion leakage and, eventually, cell death (14). The formation of MAC exerts direct cytolytic effects on target cells and releases abundant cell components, which can further activate the pattern recognition molecules and amplify the inflammatory response.

Apart from cytolysis, complement components also fulfill their roles through other mechanisms, among which the pro-inflammatory and opsonization effects are the most recognized. The small cleavage products, such as C3a, C4a, and C5a, are known as anaphylatoxins because of their ability to induce vasodilation, histamine release, and vascular permeability enhancement. These molecules also mediate the chemotaxis and activation of leukocytes (especially macrophages and neutrophils) by binding to the corresponding receptors, boosting the inflammatory response (15). The large products of serine protease cleavage, usually ending with a “b” in their names, are known as the opsonins, including C3b, iC3b, C3d, C4b, and C4d, among others (16). These fragments are deposited on the cell surfaces and mark them for phagocytosis by interacting with different complement receptors, a process known as opsonization.

### Complement Regulatory Factors

To avoid uncontrolled activation of the complement cascade and prevent its cytolytic effect on host cells, the system is also regulated by an exquisite network consisting of a variety of membrane-bound and plasma proteins. The membrane-bound complement regulators include CD46 (also known as membrane cofactor protein, MCP), CD55 (also known as decay accelerating factor, DAF), CD59, and CR1 (11). CD46, as well as its counterpart Crry in mice, acts as a cofactor for factor I (FI), which mediates the cleavage of C3b into iC3b and interrupts the assembly of C3 convertase (17). Specific ablation of Crry in tubular epithelial cells (TECs) has been reported to deteriorate the kidney IRI in mice (18). CD55 (DAF) is named on the basis of its decay accelerating activity, which impairs the activity of the C3 and C5 convertases in both the CP and AP (19). CD59 interrupts the formation of MAC by blocking the association of C9 with C5b-8 (19), preventing its cytolytic effect. Deficiency of CD55 and CD59 leads to the abnormal activation of the complement cascade and the formation of MAC on red blood cells. This results in intracellular hemolysis, which is known as paroxysmal nocturnal hemoglobinuria (20). CR1 also possesses the decay accelerating activity and acts as a cofactor of FI (21).

The complement regulatory proteins in the plasma include factor H (FH), FI, and C1 inhibitor (C1-INH), among others. As mentioned above, FI inhibits the formation of complement convertase by cleaving C3b and C4b, with the presence of other cofactors. FH possesses the decay accelerating activity toward the C3 convertase of AP and also acts as the cofactor of FI (22). C1-INH directly inactivates the serine proteases needed for the complement activation, such as C1r, C1s, and MASPs (23). Apart from these inhibitory regulatory proteins, properdin is the only positive regulator in this process, which can stabilize C3bBb and C3bBb3b from inactivation (24).

### Complement Receptors

Complement receptors are also indispensable parts of the complement system. The majority of the complement fragments bind to their receptors for corresponding functions. These receptors could be divided into two categories: the complement receptors (CR1, CR2, CR3, and CR4) and the anaphylatoxin receptors (C3aR, C5aR1, and C5aR2) (**Table 1**). Depending on where it is expressed, the functions of CR1 are dynamic (25, 26). Through binding to C3b and C4b on the immunocomplex, CR1 augments the opsonization activity of phagocytes (21). It has also been reported to modulate the activity of adaptive immune cells, such as B cells (25). However, CR1 also inhibits complement activation in several ways. Firstly, CR1 destabilizes the C3 and C5 convertases by occupying C3b and C4b. Secondly, it serves as an MBL receptor and dampens the activation of the LP. Thirdly, CR1 has been reported to possess cofactor activity in FI-mediated C3b degradation (21). CR2 is expressed on B cells and is closely related to B-cell activation and antibody production (36–38). CR3 and CR4 both belong to the integrin superfamily of adhesion proteins and share the same β-unit CD18 (27). They are responsible for opsonization by binding to their ligands C3b/iC3b (28, 39, 40) and are also involved in cell adhesion activity. Both receptors are mainly expressed on myeloid-derived cells, such as monocytes/macrophages; however, studies have shown that they also appear on certain lymphocytes (29).

**Table 1:** Expression and function of complement receptors.

	Ligand	Expression	Function
CR1 (CD35)	C3b, C4b, MBL, ficolin, C1q	Erythrocytes, neutrophils, monocytes, macrophages, dendritic cells, T cells, B cells	Destabilizes C3 and C5 convertase; acts as a cofactor of FI; inhibits LP activation; clearance of immunocomplex; opsonization; inhibits T-cell and B-cell activation (25, 26)
CR2 (CD21)	C3d, C3dg, iC3b, gp350 of Epstein–Barr virus, CD23, IFN-γ	B cells, follicular dendritic cells, T cells, epithelial cells	Co-receptor of B-cell activation; antigen uptake and presentation
CR3 (CD11b/CD18)	C3b, iC3b, C3d, C3dg, fibrinogen, ICAMs	Monocytes, macrophages, neutrophils, NK cells, T cells, microglia	Opsonization; cell adhesion; regulation of T-cell activity (27–29)
CR4 (CD11c/CD18)	C3b, iC3b, fibrinogen, ICAMs	Dendritic cells, monocytes, macrophages, neutrophils, NK cells, T cells, microglia	Opsonization; cell adhesion (27, 28)
C3aR	C3a	Granulocytes, monocytes, macrophages, parenchymal cells	Chemotaxis; granule enzyme release; increase in vascular permeability
C5aR1	C5a	Neutrophils, monocytes, macrophages, parenchymal cells	Chemotaxis; granule enzyme release; increase in vascular permeability (13, 30)
C5L2	C5a, C5a-desArg	Granulocytes, monocytes, macrophages, parenchymal cells	Pro-inflammatory/anti-inflammatory functions reported (31, 32)
CRIg	C3b, iC3b	Kupffer cells, macrophages	Opsonization; regulation of T-cell response (33–35)

MBL, mannose-binding lectin; FI, factor I; LP, lectin pathway; ICAMs, intercellular adhesion molecules; NK, natural killer.

Anaphylatoxin receptors, as the name suggests, bind to the anaphylatoxins C3a and C5a for proper functions. C3aR and C5aR1 are expressed mainly on immune cells, but are also found on other parenchymal cells, such as TECs (41). Ligation of C3aR and C5aR1 mediates neutrophil and macrophage chemotaxis and orchestrates the inflammation process (31, 42, 43). However, the role of C5aR2 is more complicated (44). C5aR2 was first discovered as a decoy receptor for C5a and therefore inhibits the pro-inflammatory C5a–C5aR1 signaling. However, studies have shown that the activation of C5aR2 also favors inflammatory response in certain occasions, for example, in the context of kidney IRI (45, 46). Moreover, recent findings in the intracellular complement system indicated the regulatory role of C5aR2 in T-cell metabolism (16), suggesting that C5aR2 acts more than a decoy receptor. Therefore, further investigation is needed to define its role in different disease models and cell types.

Taken together, the complement system was first discovered as part of the innate immune response against pathogens. Over the past decades, growing evidence in this field has greatly enriched the complement family. Also, our understanding toward complement activation has advanced beyond pathogen clearance and host defense. Numerous studies showed that complement activation is also involved in the recognition and clearance of injured host cells, therefore maintaining homeostasis. This process is achieved through the recognition of endogenous damage-associated molecular patterns (DAMPs). Moreover, various complement components and receptors bridge the innate and adaptive immune systems through contributing to the regulation of T cells and B cells.

## Involvement of Complement Activation in Kidney IRI

### Complement Pathway Triggered in Kidney IRI

IRI is the inevitable process during kidney transplantation, which predisposes kidney allograft to DGF and rejection. The involvement of the complement system in IRI is no new concept and has been extensively studied in animal models in the past decades. Although the majority of the complement components circulating in the serum are synthesized in the liver, they can also be produced by kidney parenchymal cells, such as TECs. These locally generated complement factors play a more critical role in kidney IRI than the circulating ones (3, 47, 48). This was demonstrated by the fact that the kidney isograft from *C3*-deficient mice showed only mild reperfusion damage when transplanted into *C3*-positive recipient mice, in which the complement components could still be constantly produced by the recipient’s liver (49). Inhibition of the expression of C3 with small interfering RNA (siRNA) in kidney could also ameliorate IRI (50, 51). As mentioned above, the cleavage cascade of the complement system can be triggered by three different pathways. Earlier studies indicated that the AP might be predominant during kidney IRI (52, 53). This arose from the observation that pharmacological or genetic ablation of FB, which abolished the AP convertases, ameliorated kidney IRI (7, 46, 54). On the other hand, inhibition of FH, the negative regulator of the AP, aggravated kidney injury (22, 55). More recent studies have turned their attention to the activation of the LP in kidney IRI since DAMPs exposed on kidney parenchymal cells were ideal activators of the LP (56). The inhibition of MBL, the LP initiator, alleviated the pathological injury and the inflammatory response induced by IRI. Studies have shown that the binding of MBL to the cell surface of TECs and the following internalization led to direct cell death in tubular cells, aggravating kidney injury (57). Moreover, mouse kidney allografts with MASP-2 deficiency also showed better outcomes after transplantation through LP inhibition (8). Recent findings have also highlighted the role of a novel LP initiator, collectin-11, which is a soluble lectin molecule expressed in the kidney. Collectin-11 binds to the fucosylated ligand exposed at the ischemic sites and activates the LP (3). Exogenous delivery of l-fucose, a collectin-11 ligand, protected against kidney IRI through blocking the downstream complement activation initiated by collectin-11 (9, 58). Taken together, the AP and LP are the predominant pathways triggering the complement cascade during kidney IRI, while counteracting these two pathways could effectively ameliorate complement activation and the inflammatory response, therefore preserving the integrity of the kidney.

### Involvement of Complement Regulators in Kidney IRI

Complement regulators are also involved in kidney IRI. Mice with genetic deficiency of CD55 and CD59 were more susceptible to IRI (19), while the overexpression of human CD55 and CD59 proteins in mice reduced the complement fragment deposition in the kidney and yielded better renal function (59). Deficiency of Crry, the mouse CD46 homologue, was found to be associated with aggravated IRI outcomes (18, 53). However, complement activation is not absolutely without merit in kidney IRI. A recent study has shown that the inhibition of properdin, which stabilizes the C3 convertase of the AP, led to aggravated renal function and histological changes in the kidney subjected to IRI (24). Deficiency of properdin ameliorated the complement activation during IRI. Meanwhile, it impaired the opsonization effect and the phagocytosis activity of TECs, dampened the clearance of apoptotic cells, and amplified inflammation (24).

### Involvement of Complement Receptors in Kidney IRI

Anaphylatoxin receptors are also involved in the development of kidney IRI. C3aR and C5aR1 are expressed on both infiltrating immune cells and in kidney parenchymal cells, and their expressions were elevated upon IRI (60, 61). The inhibition of either receptor reduced the expressions of chemokines and cytokines and decreased the infiltration of inflammatory cells, therefore protecting against kidney injury (61–63). Genetic ablation of these receptors conferred similar results (42, 64). Recent evidence has shown that, aside from attracting inflammatory cells such as macrophages and neutrophils (32), the activation of C5aR1 was also associated with cellular senescence within the kidney (65). Analysis of the DNA methylation in C5a-treated TECs showed aberrant methylation of the regions involved in cell cycle, DNA damage, and Wnt signaling pathway. Further experiments confirmed the existence of cell senescence in C5a-treated TECs and in kidney IRI *in vivo* (65). Also, C5aR1 was found to be expressed on fibroblasts and was involved in fibroblast activation and proliferation after IRI (13). Therefore, anaphylatoxin receptors, especially C5aR1, exerts multifaceted effects during the development of IRI by modulating the activity of a variety of cells.

Unlike the established pro-inflammatory roles of C3aR and C5aR1, the role of C5aR2 in kidney IRI remains a mystery. C5aR2 was identified as an inactive decoy receptor for C5a when first discovered since it was incapable of coupling G protein and transducing signals. However, growing evidence has raised doubts on this notion. Studies have shown that the deficiency of C5aR2 was associated with a decreased kidney injury upon IRI (45). The reduced expression of myeloperoxidase (MPO) and the decreased activity of neutrophils might account for this protective effect (32). However, controversy still exists. Another study using *C5ar2*-deficient mice failed to show any protective effect against kidney IRI (46). In addition, unlike C5aR1, the expression of C5aR2 in TECs was not elevated upon hypoxia/regeneration treatment (43), questioning the role of C5aR2 in this scenario. Moreover, the absence of C5aR2 in a murine intestinal IRI model led to worsened ischemia injury and elevated neutrophil infiltration through augmenting C5a–C5aR1 signaling (66). These controversies might partially be attributed to the fact that the *C5ar2*-knockout mice used in these experiments were of systemic genetic deficiency. Since C5aR2 is widely expressed on a variety of cell types, the deficiency of C5aR2 on certain parenchymal cells might blur the effect of C5aR2 on immune cells. Therefore, further exploration is needed to dissect the detailed function of C5aR2 on specific cell types. These controversial results also indicated that the regulation of C5aR2 and its downstream effects are more complicated than thought. Future investigations need to establish new animal models with bone marrow chimerism or cell type-specific ablation of C5aR2 and to examine the detailed role of C5aR2 signaling in kidney IRI and in other inflammatory diseases.

### Complement Components Interact With Coagulation Factors

Complement components also interplay with the coagulation cascade during activation. These two processes both belong to the innate immune response and react rapidly upon recognition of pathogen or injury. The complement system could be activated by the coagulation factors at the initiation phase. Factor XII activated the CP, while fibrin and its fragments were found to activate MASPs, the serine proteases of the LP (67, 68). Moreover, thrombin and other coagulation factors, such as factors XIa, Xa, and IXa and plasmin, could directly cleave C5 without the presence of complement serine proteases and lead to the formation of MAC (69, 70). Instead of producing C5a and C5b, thrombin cleaved C5 at a new site and produced C5a(T) and C5b(T), and the latter formed C5b(T)-9 with an even higher cytolytic activity than C5b-9 (71). Besides cleaving complement components directly, coagulation factors could also modulate complement activation through other mechanisms. Protease-activated receptors (PARs) are a subfamily of membrane-bound G protein-coupled receptors activated by the proteolytic cleavage at their extracellular domain (72). Thrombin cleaved and activated PAR-1, PAR-3, and PAR-4. The activation of PAR-1 on dendritic cells led to the elevated expressions of C3 and complement receptors (73).

On the other hand, the activated complement cascade also modulates the coagulation process. MASP1/2 of the LP could cleave prothrombin, fibrinogen, and factor XIII into their activated state since the activation of these factors relies on a similar proteolytic mechanism (68). Studies have shown that the formation of C5a and MAC resulted in the expressions of tissue factors in endothelial cells and in neutrophils, which in turn initiated the extrinsic coagulation pathway (67). C5 was also shown to cleave prothrombin in association with CD14 in a Heme-induced thromboinflammation model (74). In IRI, the release of DAMPs and tissue factors activates the complement and the coagulation cascades. The former lyses injured cells or marks them for phagocytosis, and the latter forms clots in the tubular region. These two processes orchestrated each other at multiple stages, forming positive feedback (72, 75, 76).

### Consequences of Complement Activation in Kidney IRI

Activation of the complement system upon IRI leads to multiple pathophysiological changes within the kidney. Tubular and endothelial cells are at the front line in contact with complement fragments (1). The assembly of MAC on these cells results in direct cell lysis, which serves as an amplifier for further inflammation. A study has shown that the sublytic MAC treatment in TECs also led to actin reorganization and epithelial–mesenchymal transition, the latter of which was necessary for the development of renal fibrosis after kidney injury (14). The binding of MBL and the following internalization also induced tubular cell death (57). Moreover, complement fragments were associated with elevated NADPH oxidase enzyme activity, which produced excessive reactive oxygen species (ROS) and aggravated kidney IRI (77).

The cleavage of C3 and C5 leads to the formation of anaphylatoxins. The binding of these fragments to the corresponding receptors on kidney parenchymal cells and immune cells results in the production of inflammatory cytokines together with the influx of macrophages and neutrophils, which further amplifies the inflammatory response. Large fragments produced by complement cleavage are deposited in injured cells and mark them for immune cell opsonization and phagocytosis (33). Therefore, inflammation is in the center of complement activation. The initial purpose of complement activation and the subsequent inflammatory response is to create an immune microenvironment for the clearance of injured cells and the subsequent repair process. However, severe and persistent kidney injury leads to uncontrolled inflammation and exceeds the maximum capacity of the repair mechanism, thereby aggravating kidney injury (78).

### Complement as a Therapeutic Target of Kidney IRI in Animal Models

Since complement activation and the subsequent inflammatory response are closely related to kidney IRI, multiple strategies have been developed to counteract this injury by inhibiting complement activation in animal models. These strategies could be summarized into three categories: directly inhibiting the complement cleavage cascade, targeting complement receptors, and modulating complement regulatory proteins. C1-INH, also known as the C1 esterase inhibitor, blocks both the CP and LP by inactivating the serine proteases C1 complex and MASPs. The efficacy of C1-INH in treating kidney IRI has been confirmed in several studies (79, 80). In a pig model of kidney autotransplantation, the allograft was subjected to 60 min of warm ischemia followed by 24 h of cold ischemia. C1-INH treatment alleviated the kidney injury and improved the long-term graft outcome (79). Earlier studies tried to inhibit the expressions of C3 and C5 using an RNA interference strategy (50, 51); however, the clinical significance might be limited due to the lack of targeting efficiency and to other potential side effects. A more recent study has developed a neutralizing anti-C5 antibody, called Ergidina, to solve this problem. This antibody was coupled with the cyclic RGD (Arg–Gly–Asp) peptide, which could deliver the antibody into ischemic endothelial cells (81). The targeting efficiency was confirmed in both an animal kidney IRI model and in a surgically removed human kidney specimen subjected to cold ischemia *ex vivo* (81). Another strategy focused on the complement regulatory protein FH. The authors developed a fusion protein by conjugating the function domain of FH with CRIg, which could help deliver the fusion antibody to the complement activation site (46).

## Involvement of Complement Activation in Kidney Transplant DGF

### Complement Activation Contributes to DGF

**Figure 2:**
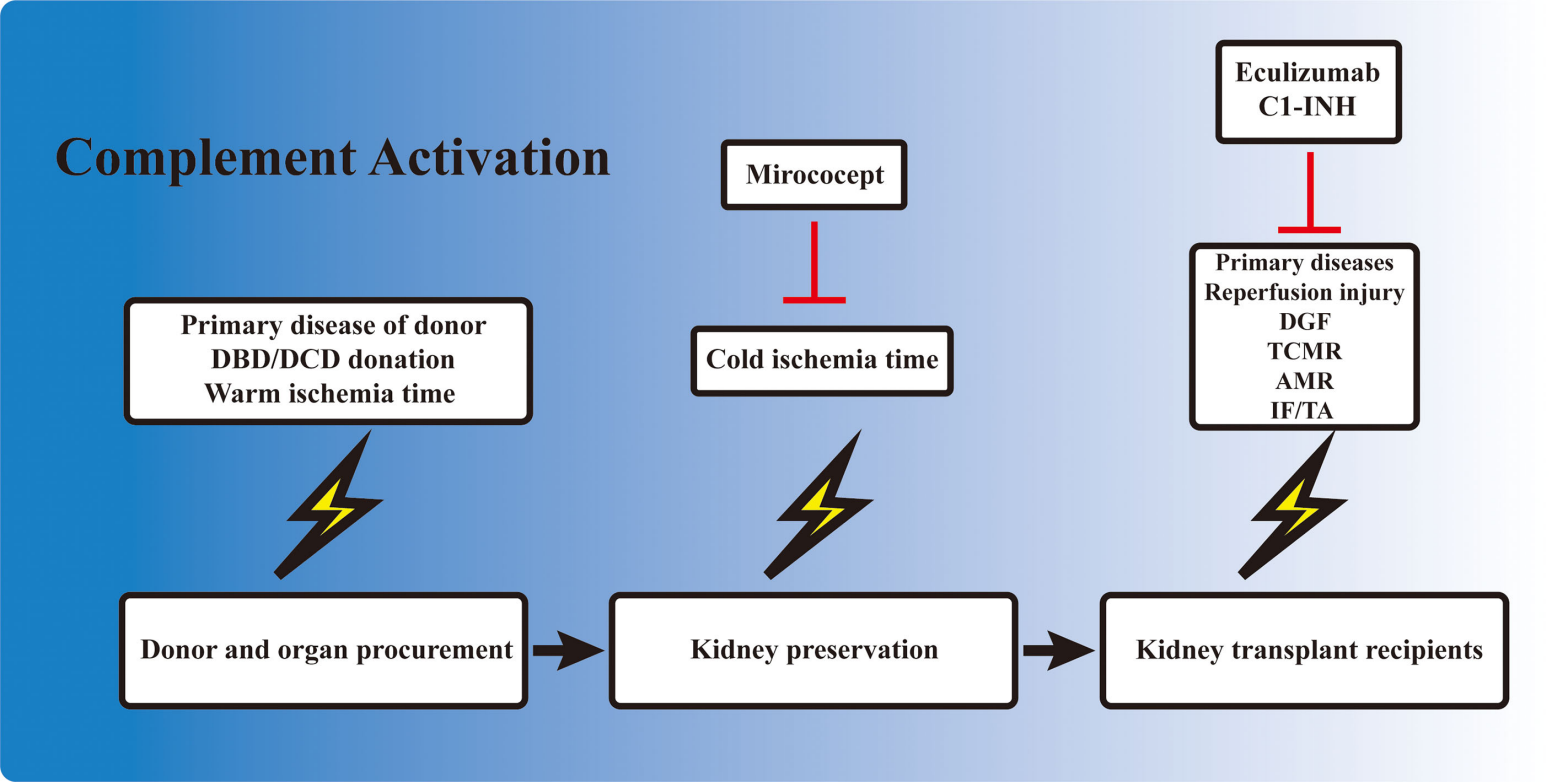
Involvement of complement activation in kidney transplantation. Activation of the complement cascade is involved before organ procurement, during *ex vivo* preservation, and also in kidney allograft recipients. The complement system is activated in deceased donors due to the unstable hemodynamics and impaired homeostasis. The kidney also undergoes a short warm ischemia period during procurement, which potentially activates the complement system. *Ex vivo* preservation of the kidney is accompanied with a cold ischemia period, which is also associated with complement gene expression. When the allograft is transplanted into the recipient, the kidney allograft first undergoes a reperfusion injury, and the subsequent delayed graft function (DGF) in certain cases. T-cell- and B-cell-mediated rejection is also activated and regulated by the complement components. Finally, the development of interstitial fibrosis/tubular atrophy (IF/TA), including the activation of fibroblasts, is associated with complement factors. Mirococept has been tested in the EMPIRIKAL trial to reduce ischemia–reperfusion injury (IRI). Clinical trials of eculizumab and C1-INH mainly focused on the reduction of DGF or the prevention/treatment of antibody-mediated rejection (AMR) in kidney transplant recipients.

Kidney IRI directly contributes to the development of DGF. However, unlike the IRI induced in animal models, during which the injury consisted of only a short period of warm ischemia followed by reperfusion, DGF in the clinical setting is also influenced by a variety of other factors. The primary disease and the physical condition of the donor, the warm ischemia time, the *ex vivo* storage period, the reperfusion during transplantation, and the subsequent alloreactive immune response could all contribute to the development of DGF, dampening allograft recovery and function. Complement activation occurs in each of these processes (**Figure 2**). Elevated complement activation has been found in kidney transplant recipients with DGF. Blood samples isolated from the renal vein during transplant reperfusion showed an increased level of soluble C5b-9 (sC5b-9) in the DGF group compared with patients with early graft function (82). Examination of kidney biopsy also revealed an increased deposition of C3d and MAC in DGF patients (46). Studies have shown that the activation of the complement system occurred prior to organ procurement and IRI (83). Transcriptomic analysis of kidney biopsy showed that the complement cascade was activated before circulation cessation and organ retrieval in brain death donors. The same phenomenon was also observed in cardiac death donors, in which the kidney underwent the first warm ischemia period. However, kidney from healthy donors did not have this effect (84). Moreover, assessment of sC5b-9 in venous blood during transplant reperfusion also indicated that sC5b-9 existed only in kidney from deceased donors, but not in those from living donors (85). Elevated AP activity was also observed in serum from deceased donors compared with their healthy counterparts (86). These reinforced the clinical observation that DGF is more prevalent in allografts from deceased donors. The presence of complement activation in deceased donors prior to organ procurement might be attributed to the unstable hemodynamics, impaired homeostasis, and the circulating DAMPs released from injured cells. These results highlighted the importance of the early inhibition of the complement system even before organ procurement, especially in deceased donors.

### Complement Factors as Biomarkers to Predict DGF

The association between complement activation and DGF makes complement fragments the ideal biomarkers for predicting allograft function and long-term outcomes. Studies have shown that the perioperative serum level of sC5b-9 had high sensitivity and specificity in predicting the 1-year allograft outcome (82). Elevated sC5b-9 levels also correlated with the prolonged duration of DGF and worsened graft function (87). In addition, attempting to predict the allograft outcome by simply genotyping specific genes, several other studies were devoted to exploring the association between the polymorphism of certain complement genes and graft function. However, the majority of these studies failed to demonstrate any correlation, which included gene polymorphism studies of the C3, C4, and MBL pathways (88, 89). This could be attributed to the limited sample size of each cohort and the inadequate follow-up period. Moreover, it has been proposed that combination of multiple complement-related gene polymorphisms might be needed to provide sufficient prediction efficiency in allograft outcomes (90).

### *Ex Vivo* Inhibition of Complement Activation to Prevent DGF

The static cold storage and machine perfusion of kidney allografts provided a perfect platform for *ex vivo* drug delivery and inhibition of the complement system. *Ex vivo* drug delivery can avoid the first pass elimination in the liver and other potential side effects. Studies have shown that *ex vivo* cold storage was associated with the upregulation of certain complement-related genes (91). This demonstrated the necessity of intervention during *ex vivo* storage to minimize complement activation. Several attempts have been made in this field and have brought some inspiring results. In a rat study, the donor kidneys were treated with either anti-rat monoclonal antibody (mAb) 18A10, a terminal complement pathway inhibitor, or TT30, a selective AP inhibitor, *ex vivo* at 4°C for 28 h before transplanting into syngeneic rat (92). Graft survival in both treatment groups improved compared with the control ones, and the 18A10-treated grafts presented better outcomes (92). This demonstrated the feasibility and efficacy of inhibiting complement activation *ex vivo*. Mirococept is a complement inhibitor derived from human CR1. It was shown to counteract IRI-induced inflammatory response in animal studies. Therefore, it was further tested in a clinical trial to reduce DGF incidence. EMPIRIKAL is a multicenter double-blind randomized case–control study that applied Mirococept to reduce DGF incidence in cadaveric kidney transplantation. Mirococept (10 mg) was given *ex vivo* during allograft preservation, and the primary endpoint was the incidence of DGF (93). However, no superiority was seen in the Mirococept treatment group; hence, the study was stopped. Further exploration in a porcine study revealed that the failure of the EMPIRIKAL study was due to insufficient Mirococept exposure. The optimal dosage was later determined to be 80 mg for pig kidney experiment, equivalent to 120 mg in a human setting, with efficient therapeutic effects and no obvious side effects (94). Further investigation is needed to explore the efficacy of *ex vivo* Mirococept delivery in kidney transplant. Other attempts in this field included perfusing and preserving the allograft in solution containing certain siRNA cocktail (95) or constructing targeted binding neutralizing antibody (81).

## Role of Complement in T-Cell-Mediated Rejection

**Figure 3:**
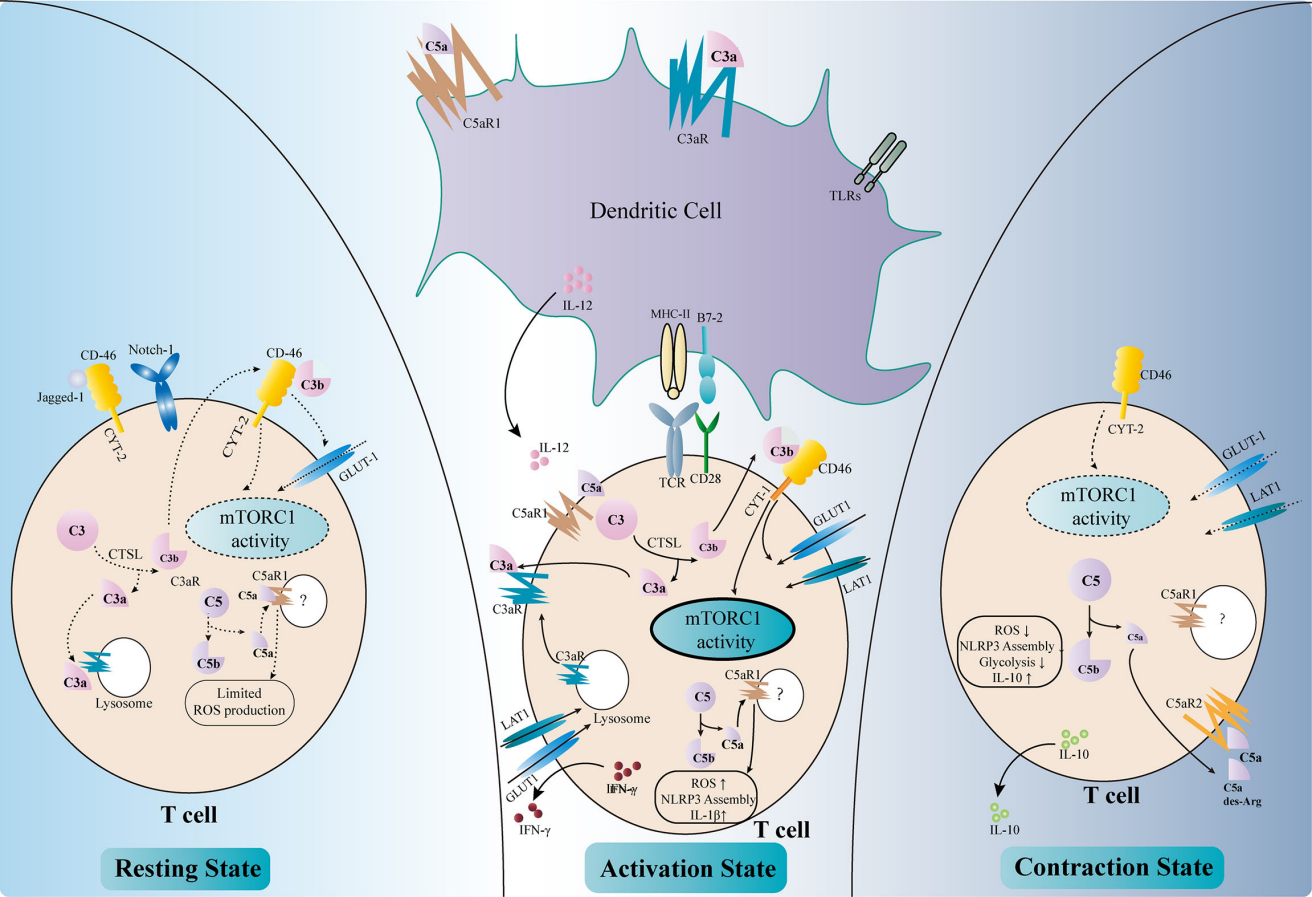
Regulation of T-cell activity by complement components. Complement factors modulate T-cell activation either directly or indirectly (*middle part of the figure*). C3aR and C5aR1 are expressed on dendritic cells (DCs). The binding of C3a and C5a, together with Toll-like receptor (TLR) signals, upregulates the expressions of MHC II, B7-2, and IL-12 and enhances the antigen presentation activity of DCs. The intracellular complement system directly regulates T-cell activity. The activation of T-cell receptors (TCRs) and co-stimulatory receptors leads to the intracellular cleavage of C3 by cathepsin L (CTSL). C3a and the C3aR transported from the lysosome to the cell surface reinforce C3a–C3aR signaling in T cells. C3b binds to CD46–CYT-1 and upregulates the nutrient transporters such as GLUT1 and LAT1. The activation of CD46–CYT-1 also enhances the activity of mTORC1, which modulates the cellular metabolism activity and promotes Th1 response. Intracellular C5a–C5aR1 signaling increases the reactive oxygen species (ROS) level in the mitochondria, therefore promoting the assembly of the NLRP3 inflammasome and the production of IL-1β. These changes lead to the production of IFN-γ necessary for Th1 response. Complement also regulates T-cell homeostasis (*left part of the figure*). In resting T cells, tonic intracellular cleavage of C3 by CTSL produces C3a and C3b. C3a binds to C3aR on lysosomes, while C3b binds to CD46–CYT-2 on the cell surface. The latter sustains a limited mTORC1 activity and the expression of GLUT-1. Intracellular C5a–C5aR1 signaling also produces limited amounts of ROS necessary for T-cell survival. In addition, CD46 could bind to Jagged-1, therefore inhibiting the Notch-1 signaling required for T-cell activation. In the contraction phase after T-cell activation (*right part of the figure*), CD46 adopts the CYT-2 isoform, identical with the resting state. This leads to decreased mTORC1 activity and declined expressions of the nutrient transporters. Instead of binding to intracellular C5aR1, C5a and its derivative C5a-desArg are secreted to the extracellular space and bind to C5aR2 on the cell surface, therefore inhibiting intracellular C5a–C5aR1 signaling. These changes result in decreased ROS production and NLRP3 activity, as well as elevated IL-10 level.

TCMR remains a major threat to graft survival after kidney transplantation. The key mechanism of TCMR is the priming and activation of T cells, including T helper (Th) cells and cytotoxic T cells (cytotoxic T lymphocytes, CTLs). The former is responsible for releasing cytokines and forms an inflammatory microenvironment, while the latter kills target cells with perforin, granzymes, and Fas ligands. The complement has long been deemed as part of the innate immunity, which is separated from the adaptive immune response. However, recent findings have indicated that the complement plays important roles in the regulation of T-cell activity, either by modulating antigen-presenting cells (APCs) or by directly influencing T cells (**Figure 3**).

### Regulation of Complement Components in Dendritic Cells

Dendritic cells (DCs) are a major type of APCs and are responsible for T-cell activation in response to stimuli (96). Capture of antigen, together with other stimulatory signals such as activation of the Toll-like receptors (TLRs), leads to the maturation of DCs and their migration to the secondary lymphoid tissue, where they present the antigen and co-stimulatory signals to elicit T-cell response. DCs rely on a variety of complement components, receptors, and regulatory proteins to fulfill their roles (97, 98). DCs with *C3* deficiency failed to stimulate T-cell response properly upon alloantigen treatment and impaired the Th1/Th2 balance, with reduced Th1 response and elevated Th2 activity (99). Moreover, deficiency of *C3* in DCs ameliorated the allograft rejection in a murine skin transplantation model (99). Further analysis revealed that *C3*-deficient DCs expressed less major histocompatibility complex (MHC) II, B7-2, and IL-12, which were necessary for inducing proper Th1 response (100). Genetic or pharmacological ablation of C3aR also elicited defective T-cell activation, suggesting that C3a–C3aR signaling was necessary for the antigen presentation activity of DCs (101). Studies have shown that the defective complement FB in DCs also led to reduced T-cell priming and activation, indicating the involvement of the AP in this setting (7, 101). On the contrary, the downregulation of DAF in DCs increased the expressions of C3a and C5a and augmented the T-cell response (102). Recent findings have shown that C5a–C5aR1 signaling was also involved in DC-induced T-cell priming and activation (10, 103). The engagement of these anaphylatoxins to their receptors drove the expressions of MHC molecules, co-stimulatory signals, cytokines, and growth factors, which were indispensable for T-cell activation and kidney allograft rejection (104). Apart from activating T cells, complement components also negatively regulate the immune response by modulating the functions of DCs. C1q, the first complement factor of the CP, was found to be associated with the differentiation of DCs and the DC-induced immune tolerance (105–107). In addition, dexamethasone treatment upregulated the expression of the complement immunoglobulin receptor (CRIg) on human monocyte-derived DCs, which was necessary for the steroid-induced negative regulation of Th1 response (108).

### Direct Effects of Complement Components in T-Cell Regulation

Anaphylatoxin receptors are also expressed on T cells and are involved during their activation. The ligation of C3aR and C5aR1 on the surface of T cells orchestrates the inflammatory response and leads to the production of IL-12 necessary for Th1 activation (102, 109). Moreover, these complement receptors also exist intracellularly within T cells and regulate T-cell responses (17). Stimulation of T-cell receptor (TCR) and the co-stimulatory receptor CD28 augments the intracellular cleavage of C3 by cathepsin L (CTSL) (110). The C3a fragments are transported to the cell membrane and bind to their corresponding receptors to further amplify the activation signal of T cells. C3aR expressed on lysosome are also transported to the cell membrane to reinforce C3a–C3aR signaling upon activation (111). Intracellular cleaved C3b could bind to CD46, a membrane-bound complement regulatory protein. CD46 was originally defined as a cofactor for FI-mediated cleavage of C3b and C4b. However, the role of CD46 in this context is far beyond a negative regulator of the complement cascade. Activated CD46 signaling promotes the alternative splicing of the CYT-1 isoform, which in turn increases the expression of the glucose transporter GLUT1 and the amino acid transporter LAT1 (112). This results in a metabolic switch toward glycolysis in T cells, which provides sufficient energy and the metabolic substrates necessary for Th1 response. CD46 also upregulates the expression of the late endosomal lysosomal adaptor, MAPK and mTOR Activator 5 (LAMTOR5), which is associated with the sensing of amino acids by mTORC1 (112). Moreover, the CD46 signals increase the nutrient influx and fatty acid oxidation activity in CD8^+^ CTLs, augmenting their cytolytic activity (113). Taken together, the increased intracellular cleavage of C3 and the activated CD46 signals result in elevated glycolysis and oxidative phosphorylation, which are essential for Th1 response and CTL activation. In accordance with these results, elevated expressions of the CD46 transcripts have been observed in the tubulointerstitial area of kidney allografts with chronic active antibody-mediated rejection (AMR), in which the Th1 response is required (114). A similar intracellular C5a–C5aR1 signaling also exists in T cells and could be amplified by CD46 signaling. Activation of intracellular C5aR1 augments ROS production from the mitochondria and promotes the assembly of the NLRP3 inflammasome (115). This further promotes the production of cleaved IL-1β and IFN-γ, which are vital for Th1 response. However, the detailed mechanisms of how C5 is cleaved intracellularly and how the cleaved fragments interact with intracellular C5aR1 remain unclear (116).

Apart from T-cell activation and Th1 polarization, complement components also participate in maintaining T-cell homeostasis (**Figure 3**). The intracellular tonic cleavage of C3 by CTSL generates C3a, which interacts with C3aR on lysosomes and results in a minor but sufficient mTOR signaling and glycolysis activity for T-cell survival (111). CD46 is alternatively spliced into the CYT-2 isoform in resting T cells and maintains the expressions of GLUT1 and Bcl-2 to avoid apoptosis (104). In addition, CD46 has been shown to bind Jagged-1, the Notch-1 ligand, therefore blocking the Notch signaling essential for T-cell activation (17). A low level of intracellular C5a–C5aR1 signaling has also been proposed to maintain T-cell homeostasis by producing limited amounts of ROS. However, the detailed mechanism remains unclear (17).

Complement components also regulate the “contraction” phase of Th1 response to resolve inflammation (**Figure 3**). Again, CD46 plays a major role in this scenario (117). CD46 turns back to the CYT-2 isoform, in accordance with the resting phase, and downregulates the expressions of GLUT1 and LAT1, therefore decreasing glycolysis and mTOR activity (16). Moreover, the anaphylatoxin C5a and its fragment C5a-desArg could be secreted to the extracellular space and suppress the ROS activity by binding to cell surface C5aR2 (16). These effects lead to the production of IL-10, which resolves the pro-inflammatory response.

Taken together, the intracellular complement components, especially the anaphylatoxin receptors and CD46 signaling, dictate T-cell homeostasis, activation, and contraction. These factors regulate the T-cell activity mainly by modulating the cellular metabolic activity. However, evidence of intracellular complement activation is still limited in the field of organ transplantation, which might be partially attributed to the versatile function of CD46. Since CD46 also acts as a cofactor of FI-mediated cleavage of C3b, certain CD46 polymorphism was found to be associated with reduced acute rejection and better allograft survival in kidney transplant recipients (118). Transgenic expression of human CD46 was also a promising strategy to attenuate hyperacute rejection in xenograft experiments (119, 120). Therefore, further investigations in this field need to focus on the versatile function of CD46, and targeted intervention of intracellular complement signaling might be a promising therapy to attenuate T-cell activation and alleviate allograft rejection.

## Role of Complement in Antibody-Mediated Rejection

AMR remains the leading cause of graft loss after kidney transplantation, and it lacks efficient treatment. Knowledge of complement activation in AMR arose from the findings of complement-dependent cytotoxicity (CDC) (121) and the deposition of C4d in peritubular capillaries in kidney allografts (122). Complement components regulate the development of AMR and also participate in AMR-mediated allograft injury.

The complement receptor CR2 participates in B-cell-mediated humoral response. The binding of C3d-opsonized antigen to CR2 on B cells promotes B-cell activation and antibody production by lowering its activation threshold (123). CR2 expressed on follicular DCs retains C3d-opsonized antigens within the germinal center, which is essential for memory B-cell maintenance (104). In addition, CR2 signaling is associated with the negative selection of self-reactive B cells and the positive selection of B-1 cells (17). The intracellular complement system, which is vital for T-cell homeostasis and activation, is also observed in B cells (17). However, the involvement of the intracellular complement activation in B-cell activation and antibody production awaits further investigation.

Complement is also associated with AMR-mediated allograft injury. Donor-specific antibodies (DSAs), including anti-ABO and anti-HLA antibodies, bind to endothelial cells during AMR and enable the engagement of C1q, which initiates the CP. Activation of the complement cascade and the assembly of MAC lead to endothelial cell lysis and dampen the vasculature integrity of allografts, a process known as CDC. MAC also initiates the pro-inflammatory signaling in endothelial cells (10). Together with the anaphylatoxins C3a and C5a produced during the cleavage cascade, it reinforces this inflammatory microenvironment (124). In addition, activation of the complement system promotes the adaptive immune response. Studies have shown that the antibody-induced MAC on endothelial cells enhanced the alloreactive T-cell response and increased the production of IFN-γ, which was essential for Th1 polarization and further amplified the humoral response (124). In addition, the assembly of MAC also leads to the production of tissue factors in endothelial cells, which could initiate the coagulation cascade (125). This partially explains the presence of thrombotic microangiopathy in certain AMR cases.

Deposition of C4d has been deemed as one of the diagnostic criteria of AMR. However, emerging pieces of evidence are questioning the clinical significance of C4d in this scenario. This arose from the observation that kidney transplant recipients without AMR could also present C4d deposition in allograft biopsy, while the absence of C4d was not sufficient to exclude AMR (126, 127). A retrospective study found that, although C4d-positive AMR patients presented clinical manifestations earlier and more frequently when compared with C4d-negative AMR patients, the 1- and 2-year graft survival rates post-AMR diagnosis were similar between the two cohorts (127). In addition, the clinical characteristics and the risk of graft loss were similar between the two cohorts despite the absence of C4d (127). In fact, in the current Banff criteria, C4d is no longer the prerequisite for AMR diagnosis, and “C4d staining without evidence of rejection” has been included as a subtype of antibody-mediated changes (128). The declining importance of C4d gives way to the DSA assay measuring complement binding activity (129). C3d-binding DSAs in AMR patients were associated with increased graft loss, deteriorated allograft function, and higher DSA mean fluorescence intensity (MFI) (130). Another study in non-sensitized pediatric kidney recipients reinforced the value of C3d-binding DSAs in predicting long-term graft survival (131). DSAs could be further classified as *de novo* DSAs (dnDSAs) and preformed DSAs (pDSAs). The former are developed after transplantation and the latter exist in the recipients before transplantation. Positive C3d-binding dnDSAs were found to be associated with the presence of AMR (132, 133). A study on pDSAs indicated that half of the C3d-negative pDSAs disappeared after transplantation, while all C3d-binding pDSAs persisted despite desensitization (134). Moreover, C3d-binding pDSAs were associated with a higher risk of developing AMR (134). However, the role of C1q-binding DSAs has been controversial. Some studies showed similar or minor predictive value of C1q-binding with C3d-binding DSAs (130–132), while some highlighted that the C1q-binding capacity could only reflect the MFI of DSAs instead of predicting allograft prognosis (135).

Taken together, the activation of the complement system participates in the regulation of humoral response and plays roles in antibody-mediated graft injury. Moreover, complement fragment-binding DSAs seem a promising feature for predicting AMR and graft survival. Therefore, complement could serve as a potential therapeutic target of AMR. Several clinical trials using eculizumab or C1-INH in the prophylaxis or treatment of AMR have shown encouraging results and might provide additional choice for AMR treatment in the future (**Tables 2** and **3**).

**Table 2:** Clinical trials of eculizumab in kidney transplantation.

Identifier no.	Study start date	Phase	Purpose	Experimental treatment	Status
NCT02113891 (136)	February 2015	Phase I/II	Eculizumab therapy for subclinical antibody-mediated rejection in kidney transplantation	Eculizumab 900 mg (i.v.) every 7 days for 4 doses, a fifth 1,200-mg dose 7 days later followed by 15 maintenance doses: 1,200 mg every 14 days	Withdrawn
NCT01919346 (137)	August 2013	Phase II	Eculizumab for the prevention of delayed graft function (DGF) in kidney transplantation	Eculizumab 1,200 mg prior to reperfusion of the renal allograft and again at 900 mg 12–24 h post-transplantation	Terminated
NCT01029587 (138)	November 2009	Phase II	Eculizumab to enable renal transplantation in patients with a history of catastrophic antiphospholipid antibody syndrome	Eculizumab 1,200 mg on the day of or on the day prior to kidney transplantation and 900 mg on postoperative day 1. Then, 900 mg (i.v.) on postoperative days 8, 15, and 22, followed by 1,200 mg (i.v.) on postoperative days 29, 43, 47, 72, and 85	Completed
NCT01095887 (139)	May 2010	Phase I/II	Eculizumab to prevent antibody-mediated rejection in ABO blood group-incompatible living donor kidney transplantation	Eculizumab was given on day 0, day 1, and weekly for the first 4 weeks after transplantation	Terminated
NCT01327573 (140)	March 2011	Phase I	Eculizumab therapy for chronic complement-mediated injury in kidney transplantation	Eculizumab induction: 600 mg (i.v.) every 7 days for 4 doses, followed by 900 mg (i.v.) 7 days later	Completed
				Eculizumab maintenance: 900 mg (i.v.) every 14 days for a total of 26 weeks	
NCT00670774 (141)	March 2008	Phase I/II	Dosing regimen of eculizumab added to conventional treatment in positive cross-match living donor kidney transplantation	Eculizumab 1,200 mg 1 hour prior to surgery	Completed
				Eculizumab 900 mg on day 1 post-transplant; then eculizumab 900 mg weekly through 4 weeks post-transplant	
NCT01403389 (142)	December 2011	Phase II	A study of the activity of eculizumab for the prevention of delayed graft function in deceased donor kidney transplant	Eculizumab 1,200 mg prior to organ reperfusion	Terminated
NCT01106027 (143)	March 2010	Phase I/II	Dosing regimen of eculizumab added to conventional treatment in positive cross-match deceased donor kidney transplantation	Eculizumab 1,200 mg 1 h prior to surgery;	Terminated
				Eculizumab 900 mg on day 1 post-transplant; then eculizumab 900 mg weekly through 4 weeks post-transplant	
NCT01895127 (144)	November 2013	Phase II	Efficacy and safety of eculizumab for the treatment of antibody-mediated rejection following renal transplantation	Eculizumab 1,200 mg after biopsy-proven AMR; then 900 mg weekly for 4 doses (weeks 1–4); followed by 1,200 mg on week 5	Terminated
				Week 6: if donor-specific antibody <50% of the baseline DSA, then no further treatment; otherwise, 1,200 mg weeks 7 and 9	
NCT01399593 (145)	November 2011	Phase II	Safety and efficacy of eculizumab to prevent AMR in living donor kidney transplant recipients requiring desensitization	Eculizumab 1,200 mg prior to allograft transplantation; eculizumab 900 mg (days 1, 7, 14, 21, and 28), and eculizumab 1,200 mg (weeks 5, 7, and 9)	Terminated (146)
NCT01567085 (147)	August 2012	Phase II	Safety and efficacy of eculizumab in the prevention of AMR in sensitized recipients of a kidney transplant from a deceased donor	Eculizumab 1,200 mg prior to kidney allograft reperfusion;	Completed
				Eculizumab 900 mg on post-transplant days 1, 7, 14, 21, and 28;	
				Eculizumab 1,200 mg on post-transplant days 35, 49, and 63	
NCT02145182 (148)	August 2014	Phase II/III	Prevention of delayed graft function using eculizumab therapy (PROTECT Study)	Eculizumab given on the day of transplantation, then 18–24 h later	Completed
NCT01756508 (149)	September 2012	Phase II	Eculizumab for the prevention and treatment of kidney graft reperfusion injury	Eculizumab 1,200 mg/m^2^ given 1 h before graft reperfusion	Completed

AMR, antibody-mediated rejection; DSA, donor-specific antibody.

**Table 3:** Clinical trials of C1-INH in kidney transplantation.

Identifier no.	Study start date	Phase	Purpose	Experimental treatment	Status
NCT01035593 (150)	December 2010	Phase II	Recombinant human C1 inhibitor for the treatment of early AMR in renal transplantation	Plasmapheresis + 100 mg/kg IVIG every other day × 5 treatments plus rhC1Inh 100 U/kg (i.v.) daily × 7 consecutive days	Withdrawn
NCT01134510 (151, 152)	August 2011	Phase I/II	Prevent complement-dependent, AMR post-transplant in highly HLA-sensitized patients	C1 esterase inhibitor 20 U/kg twice weekly × 4 weeks	Completed
NCT02134314 (153)	May 2014	Phase I/II	C1INH inhibitor preoperative and post-kidney transplant to prevent DGF and IRI	C1 esterase inhibitor 50 U/kg on day of transplant and another dose at 24 h postoperatively	Completed
NCT02936479 (154)	October 2016	Phase II	C1 inhibitor (INH) for refractory antibody-mediated renal allograft rejection	C1-INH (Berinert)	Completed
NCT03221842 (155)	November 2017	Phase III	C1 esterase inhibitor as add-on to standard of care for the treatment of refractory AMR in adult renal transplant recipients	C1 esterase inhibitor	Terminated (156)
NCT02547220 (157)	May 2016	Phase III	Treatment of acute AMR in participants with kidney transplant	CINRYZE 5,000 U on day 1 and 2,500 U on days 3, 5, 7, 9, 11, and 13	Terminated
NCT01147302 (158)	August 2011	Phase II	Use of the C1 esterase inhibitor (human) in patients with acute AMR	C1 esterase inhibitor 5,000 U (not to exceed 100 U/kg) on day 1, followed by 2,500 U (not to exceed 50 U/kg, i.v.) on days 3, 5, 7, 9, 11, and 13	Completed (159)
NCT04696146 (160)	March 2021	Phase I/II	Assessing the safety and efficacy of preoperative renal allograft infusions of C1 inhibitor (Berinert^®^) (human, C1INH) *vs*. placebo administration in recipients of a renal allograft from deceased high-risk donors and its impact on DGF and IRI	Berinert 500 U	Recruiting
NCT03791476 (161)	June 2019	Phase I	RUCONEST^®^ as a therapeutic strategy to reduce the incidence of DGF	rhC1INH 100 U/kg intraoperatively, followed by 50 U/kg every 12 h × 2 = total of 3 doses (200 U/kg)	Recruiting
NCT02435732 (162)	December 2020	Phase I	CINRYZE as a donor pretreatment strategy in kidney recipients of KDPI > 60%	CINRYZE 200 U/kg (i.v.) single dose	Not yet recruiting

IVIG, intravenous immune globulin; HLA, human leukocyte antigen; DGF, delayed graft function; IRI, ischemia–reperfusion injury; AMR, antibody-mediated rejection; KDPI, kidney donor profile index.

## Complement Activation in Chronic Allograft Injury

Chronic allograft nephropathy, now known as interstitial fibrosis/tubular atrophy (IF/TA), is the consequence of maladaptive repair after allograft injury. A variety of causes, including but not limited to IRI, drug toxicity, infection, and rejection, could all lead to the progression of IF/TA (78). The development of IF/TA usually involves the presence of tubular injury, unresolved inflammation, and microvasculature refraction (78). All these pathological changes lead to myofibroblast activation and the production of extracellular matrix, which forms a scar in the kidney and causes gradual loss of allograft function. Activation of the complement system leads to a direct cytolytic effect on tubular and endothelial cells, which impairs tubular function and the vasculature integrity. Anaphylatoxins attract immune cell infiltration, forming an inflammatory microenvironment that favors the development of IF/TA. Abundant evidence has confirmed the role of complement in the development of renal fibrosis in animal models (12, 13, 163). However, the majority of these studies only focused on the fibrotic phenotype of the kidney, while evidence for the regulation of fibroblast activity by complement components is still limited. Peng et al. demonstrated the presence of C5aR1 on resting primary kidney fibroblast and the elevation of its expression upon hypoxia (13). Moreover, C5a treatment led to increased proliferation and mesenchymal activation in resting fibroblast (13). Scattered pieces of evidence in other fields also reinforce the role of complement components in the regulation of fibroblast activity. Earlier studies showed that C5a–C5aR2 signaling was required for fibroblast functions (164). In a diabetic kidney disease model, C3aR and C5aR1 activation induced the mesenchymal transition in endothelial cells, a process closely related to fibroblast activation (165). Treatment of human primary lung fibroblasts with C3a and C5a stimulated their mesenchymal activation and the production of extracellular matrix (166). Studies in pulp fibroblasts also showed that anaphylatoxin receptor signaling was involved in the activation of fibroblasts after injury (167–169). CD10^+^C5aR2^+^ cancer-associated fibroblasts were associated with the inflammatory microenvironment favoring tumor stemness, which was also required for the development of IF/TA (170, 171). Recent research in arthritis has highlighted the role of intracellular C3aR signaling in synovial fibroblasts. The activation of C3aR led to increased metabolic activity in these cells, which was mediated by the elevated mTOR signaling and HIF-1α production. The NLRP3 inflammasome was also induced upon C3aR activation, enhancing local inflammation and aggravating arthritis (172). This is in accordance with the findings of the intracellular complement modulation in T cells. Since fibroblasts are a highly heterogenic cell population, the detailed involvement and the mechanism of complement activation in fibroblast activity urge further investigations.

## Targeting Complement Activation in Kidney Transplant Recipients

Apart from intervention before and during organ procurement, targeting the complement system in allograft recipients is also a promising strategy. Several complement inhibitors were developed and underwent clinical trials in kidney transplant recipients, among which eculizumab and the C1 esterase inhibitor are the most promising ones. Eculizumab is a recombinant humanized hybrid IgG2/IgG4 monoclonal antibody that targets C5, the key molecule of complement activation. The binding of eculizumab to C5 prevents its cleavage by serine proteases, therefore inhibiting the production of C5a and the assembly of MAC (125). Eculizumab has been approved for the treatment patients with paroxysmal nocturnal hemoglobinuria (PNH) and atypical hemolytic uremic syndrome (aHUS), in which aberrant activation of the complement system is involved in the disease progression. Studies have shown that the prophylactic treatment of eculizumab enabled successful kidney transplantation in patients with aHUS and was efficient for improving aHUS recurrence after transplantation (173, 174). Eculizumab treatment was also beneficial for successful renal transplantation in patients with antiphospholipid antibody syndrome or C3 glomerulopathy (175–177). Eculizumab treatment has been suggested for the prevention or treatment of AMR in several clinical trials (**Table 2**). A recent single-arm trial examining the safety and efficacy of eculizumab in preventing AMR in sensitized recipients (NCT01567085) has shown encouraging outcomes. However, controversy in this field still exists. NCT01895127, a study that evaluated the efficacy of eculizumab treatment in kidney transplant recipients with biopsy-proven AMR, failed to demonstrate the superiority of eculizumab compared with standard plasmapheresis and immunoglobin treatment. These controversies might be ascribed to the small study cohort and the insufficient eculizumab exposure in these studies. Several clinical trials in this field have been terminated for poor enrollment, or were conducted in a small cohort, limiting the confidence in these trials. Moreover, the combination of eculizumab with the current standard therapy might provide better clinical outcomes. Eculizumab has also been tested to prevent IRI or DGF in kidney allograft recipients. Disappointingly, a recent published result has shown that the DGF incidence and the early and late posttransplant graft functions were similar between the eculizumab-treated group and the control group (178).

C1-INH, an endogenous complement regulatory protein, is also extensively studied in kidney transplantation recipients. C1-INH binds to and inactivates the initial serine proteases of the complement cascade, including C1r, C1s, and MASPs. Compared with eculizumab, which targets the cleavage of C5, C1-INH inhibits both the CP and LP at the initiation stage, therefore also eliminating the production of the C3 and C4 fragments (179). C1-INH was mostly given perioperatively to protect against IRI and DGF in animal studies and clinical trials (**Table 3**). A recent non-human primate kidney transplantation study has suggested that treatment of C1-INH in DBD (donation after brain death) donors prevented the development of DGF and improved allograft function (180). A randomized clinical trial using two doses of C1-INH perioperatively also showed encouraging outcomes (181). In brief, 70 kidney recipients were equally randomized to either the C1-INH treatment group or the control group. C1-INH was given at 50 U/kg twice. The first dosage was given intraoperatively and the second at 24 h post-operation. Although the incidence rates of DGF were similar between the two groups, C1-INH treatment led to fewer dialysis session 2–4 weeks after transplantation. The C1-INH treatment group also presented better renal function 1 year post-transplantation compared with the control counterpart (181). Follow-up of the same study revealed that C1-INH treatment decreased allograft loss and improved the estimated glomerular filtration rate (eGFR) even 3 years after transplantation (182). Several upcoming clinical trials evaluating the efficacy of C1-INH in improving DGF are underway and might provide further evidence for the use of C1-INH in kidney transplantation (**Table 3**). C1-INH was also used to prevent or treat AMR in kidney transplant recipients (23, 151, 183). A double-blind randomized trial evaluated the use of C1-INH together with standard therapy in patients with AMR (NCT01147302) (159). Treatment with C1-INH yielded a trend toward sustained improvement of allograft function. In addition, C1-INH decreased the possibility of developing transplant glomerulopathy compared with the control group (159). In all, the results from recent clinical trials demonstrated that C1-INH might be a feasible treatment for patients with AMR.

Apart from eculizumab and C1-INH, several other complement inhibitors have been developed and have undergone clinical trials for the treatment of PNH and aHUS. Multiple monoclonal antibodies, such as crovalimab, pozelimab, tesidolumab, and ravulizumab, inhibit complement activation through targeting C5 (184–187). These antibodies, similarly to the previously mentioned eculizumab, bind to C5 and prevent the downstream activation of the complement cascade. Ravulizumab, also known as Ultomiris, has already been approved to for the treatment of PNH and aHUS in the USA (188). Nomacopan, formerly known as Coversin, is a small protein that also prevents the cleavage of C5 by the C5 convertase (189). Cemdisiran represents another strategy that suppresses the liver-produced C5 with RNA interference (190). Despite that the majority of these complement inhibitors target C5, pegcetacoplan is the first and only targeted C3 therapy for the treatment of PNH (191). Pegcetacoplan is a PEGylated peptide that binds to C3 and its fragment C3b, eliminating both the cleavage of C3 and the opsonization effect of C3b (191). A recent study has suggested that pegcetacoplan showed superior therapeutic effect than eculizumab in the treatment of PNH (192). This was probably attributed to the early inhibition of the complement cascade by pegcetacoplan. Several orally administered complement inhibitors have also been developed. Vemricopan and danicopan inhibit FD in the AP (193), while iptacopan targets FB (194). In addition, avacopan, a selective C5aR inhibitor, has been approved for the treatment of anti-neutrophil cytoplasmic antibody (ANCA)-associated vasculitis and has been tested in patients with aHUS (195). Future investigations of these complement inhibitors in kidney transplant recipients might provide additional choices for the prevention and treatment of various complications and improve allograft outcomes.

## Conclusion and Future Perspectives

Complement components are widely expressed in kidney parenchymal cells and in immune cells. Activation of the complement system is involved throughout the whole process of kidney transplantation. Uncontrolled complement cleavage cascade in deceased donors and IRI predispose the allograft to DGF. During this process, the complement system serves predominantly as part of the innate immune response through assembling MAC and orchestrating the inflammatory response. Growing evidence indicates that the complement components could also bridge the innate and adaptive immune responses and are involved in the development of TCMR and AMR after kidney transplantation. The emerging intracellular complement system plays the central role in this scenario, especially in the regulation of T-cell activity and homeostasis. The involvement of the complement system in kidney transplantation makes it an ideal predictive tool and a promising therapeutic target in the clinical setting. Results from animal models and clinical trials suggested that interfering complement activation before organ procurement, during kidney preservation, and in kidney transplant recipients were all plausible strategies to improve allograft outcomes. Future investigations in this field need to focus on the intracellular complement components and those locally produced in the kidney and to further improve the targeting efficiency of these therapies.

## Author Contributions

WQ conceived this review. RQ collected and analyzed literatures and drafted the manuscript. WQ helped revise the language. All authors read and approved the final manuscript.

## Conflict of Interest

The authors declare that the research was conducted in the absence of any commercial or financial relationships that could be construed as a potential conflict of interest.

## Publisher’s Note

All claims expressed in this article are solely those of the authors and do not necessarily represent those of their affiliated organizations, or those of the publisher, the editors and the reviewers. Any product that may be evaluated in this article, or claim that may be made by its manufacturer, is not guaranteed or endorsed by the publisher.
